# Patient-reported symptoms and interest in symptom monitoring in HCC treated with locoregional therapies: A qualitative study

**DOI:** 10.1097/HC9.0000000000000315

**Published:** 2023-11-06

**Authors:** Andrew M. Moon, Sarah Cook, Rachel M. Swier, Hanna K. Sanoff, Michael D. Kappelman, Lynne I. Wagner, A. Sidney Barritt, Amit G. Singal, Neil D. Shah, David M. Mauro, Ted K. Yanagihara, David A. Gerber, Michael W. Fried, Cristal Brown, Myra Waheed, Randall Teal, Donna M. Evon

**Affiliations:** 1 Division of Gastroenterology and Hepatology, University of North Carolina, Chapel Hill, North Carolina, USA; 2 Lineberger Comprehensive Cancer Center, University of North Carolina, Chapel Hill, North Carolina, USA; 3 Virginia Commonwealth School of Medicine, Richmond, Virginia, USA; 4 University of North Carolina School of Medicine, Chapel Hill, North Carolina, USA; 5 Division of Oncology, University of North Carolina, Chapel Hill, North Carolina, USA; 6 Department of Pediatrics, Division of Pediatric Gastroenterology, University of North Carolina, Chapel Hill, North Carolina, USA; 7 Department of Health Policy and Management, University of North Carolina, Chapel Hill, North Carolina, USA; 8 Division of Digestive and Liver Diseases, University of Texas Southwestern Medical Center, Dallas, Texas, USA; 9 Division of Vascular Interventional Radiology, University of North Carolina, Chapel Hill, North Carolina, USA; 10 Department of Radiation Oncology, University of North Carolina, Chapel Hill, North Carolina, USA; 11 Department of Surgery, University of North Carolina, Chapel Hill, North Carolina, USA; 12 Department of Medicine, University of Texas at Austin, Austin, Texas, USA; 13 Connected Health Applications and Interventions (CHAI) Core, University of North Carolina, Chapel Hill, North Carolina, USA

## Abstract

**Background:**

Patient-reported outcomes (PRO) measures relevant to domains most important to patients with HCC who received locoregional therapies are needed to advance patient-centered research. Furthermore, electronic PRO monitoring in clinical care has been shown to reduce hospitalizations and deaths in patients with other cancers. We conducted a qualitative study among patients with HCC who recently received locoregional therapies to (1) identify common and distressing posttreatment symptoms to prioritize PRO domain selection and (2) gauge interest in an electronic PRO symptom monitoring system.

**Methods:**

We performed semi-structured telephone interviews among adult patients who received locoregional therapies (median of 26 days after treatment) for treatment-naïve HCC at a single tertiary care center. Interviews were conducted until thematic saturation was reached. Qualitative content analysis was conducted to identify emerging themes and sub-themes.

**Results:**

Ten of 26 patients (38%) reported at least 1 symptom before treatment. In contrast, all participants (n = 26) with recently treated HCC reported at least 1 posttreatment physical symptom, with the most common being appetite loss (73%), fatigue (58%), abdominal pain (46%), and nausea (35%). Most participants (77%) stated they saw potential benefits in posttreatment ePRO symptom monitoring.

**Conclusions:**

Posttreatment symptoms after HCC locoregional therapies are common and often severe. These data can inform and prioritize PRO domain selection. Patients are interested in ePRO monitoring to monitor and proactively address posttreatment symptoms. Given the clinical benefits in patients with metastatic cancers, ePRO monitoring warrants investigation in patients with HCC.

## Graphical abstract

**Figure:**
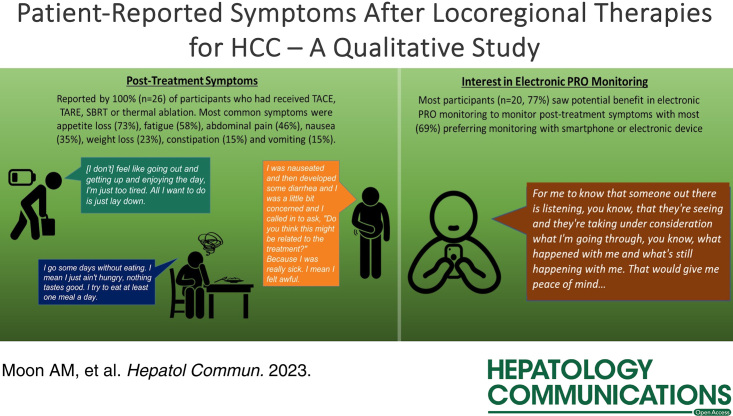

## INTRODUCTION

HCC is expected to become the third leading cause of cancer-related death in the United States by 2035.^1,2^ Fewer than 50% of patients with newly diagnosed HCC are eligible to receive potentially curative surgical treatments.^3–5^ Patients with liver-localized, unresectable HCC may be candidates for locoregional therapies (LRTs), including thermal ablation, transarterial chemoembolization (TACE), transarterial radioembolization (TARE), and stereotactic body radiation therapy (SBRT). With few exceptions, LRTs are not curative-intent procedures, meaning they do not result in life expectancy similar to those without HCC.^6^

Patient-reported outcomes (PROs) that assess patients’ experiences with cancer and cirrhosis treatments are increasingly being identified as important endpoints by patients, clinicians, and regulators,^7–12^ yet little is known about patients’ posttreatment experiences and symptoms for patients with HCC following LRT. A better understanding of posttreatment symptoms after LRTs is needed to inform the selection of PROs most relevant for this population. Once selected, PROs can be incorporated into patient-centered outcomes research and enhance clinical care by informing patients, caregivers, and clinicians of treatment decisions and preparing patients for post-LRT recovery.

There is a growing literature on the role of PROs in HCC. A recent scoping review identified several physical symptoms that were identified through prior qualitative work.^12^ A number of PRO domains were identified in this work, including physical symptoms such as body pain, gastrointestinal symptoms (abdominal pain, bloating, diarrhea, anorexia), fatigue, jaundice, rash, vertigo, weakness, and dizziness.^12^ Additionally, a multinational survey reported that side effects related to HCC treatments are common, including > 60% reporting abdominal pain and fatigue, 60% reporting absenteeism from work, 37% with inability to carry out daily activities, and 28% experiencing negative impacts on relationships with family/caregivers.^13^

Accumulating evidence on the use of PROs also exists in cancer care^14^ and, recently, the PRO-TECT trial, a multicenter randomized trial, demonstrated that electronic PRO monitoring among patients with metastatic cancer improved patient-reported physical function, symptom control, and HRQOL.^15^ A published single-center trial demonstrated significantly improved overall survival rates in the PRO monitoring group (31 mo) compared to the usual care arm (26 mo).^16^ Symptom monitoring with PROs may be particularly important in people with cirrhosis and HCC, given that underlying cirrhosis is present in 80%–90% of individuals diagnosed with HCC. Patients with cirrhosis experience a high baseline rate of acute care utilization that could be exacerbated by LRT for those with concurrent HCC. Research in patients with metastatic cancers has shown that electronic PRO monitoring significantly improves outcomes.^15–18^ Most published qualitative studies on symptoms in HCC were focused on advanced-stage disease and were not designed to assess symptoms post-LRT.^19–22^ Furthermore, no studies to date have explored patients’ interest in a remote PRO monitoring system to proactively evaluate and respond to post-LRT symptoms.

Therefore, we conducted a qualitative study using semi-structured individual interviews with adult patients who had recently undergone LRT for HCC. The current analysis aimed to (1) identify common and distressing posttreatment symptoms in patients’ own words and (2) gauge patient interest in an electronic PRO symptom monitoring system.

## METHODS

### Study design

This was a single-center qualitative study using a narrative research approach. Qualitative interviews and analyses were carried out by the University of North Carolina (UNC) Connected Health Applications and Interventions (CHAI) Core, an NIH-funded core that conducts qualitative research and e-health intervention development. The investigative team was comprised of physicians in the fields of hepatology, oncology, interventional radiology, radiation oncology and transplant surgery, qualitative research experts, and experienced qualitative analysts. All research was conducted in accordance with the Declarations of Helsinki and Istanbul, institutional review board approval was provided by the UNC institutional review board (#20-2840), and patients provided written consent before the initiation of any study procedures.

### Study sample

Inclusion criteria were: age ≥ 18 years, HCC diagnosis based on American Association for the Study of Liver Diseases criteria,^23^ plan to receive thermal ablation, TACE, TARE or SBRT, and English-speaking. We excluded patients who were receiving re-treatment of a previously treated lesion, given that this may affect posttreatment symptoms. Patients were identified through multidisciplinary tumor board and HCC clinics, including the multidisciplinary HCC clinic. Attempts were made to enroll all consecutive patients identified who met inclusion/exclusion criteria. Participants were interviewed 2–6 weeks after receiving or, in the case of SBRT (which requires multiple treatments over several days), starting LRT.

### Data collection procedures

A total of 26 in-depth interviews were conducted between September 2021 and August 2022. Enrollment was stopped when thematic saturation was achieved. The interviews were conducted by 3 trained qualitative researchers who are part of the CHAI Qualitative Research Group, which is a shared-resource core for the UNC Lineberger Comprehensive Cancer Center. The CHAI Qual Group works with a wide range of different clinical investigators across a variety of oncologic departments (eg, current projects include kidney, bladder, liver, lung, breast, testicular, and pancreatic cancers). The CHAI Qualitative Research Group are experts on the technical and methodological aspects of qualitative research and collaborate on funded projects with investigators who are experts in the topical or content areas in which they practice.

Interviews were completed over the telephone, lasted ~45–60 minutes, and were digitally recorded. The interviewers followed an interactive, semi-structured interview guide (Appendix A in Supplemental Materials, http://links.lww.com/HC9/A644). The interview guide was developed in a collaborative manner between the investigators and the qualitative experts at CHAI Core. The interview guide was pilot-tested among the qualitative interviewers but not formally tested with patients with HCC prior to data collection. However, iterative changes were made to the semi-structured interview guide based on interviewer experience and feedback during the first few interviews. Participants received a $50 gift card for completing the interview.

### Data analysis

All interviews were recorded, transcribed, and de-identified. Interview transcripts were imported into Dedoose version 9.0.86 (Los Angeles, CA, www.dedoose.com. We developed a codebook based on the research questions and notes taken during data collection. The initial codebook was pilot-tested by 4 qualitative researchers who independently coded transcripts, which led to the fine-tuning of coding definitions and revising decision rules on when to apply codes. Emerging themes and discrepancies were captured and reconciled by discussion and consensus. This process continued until replicability of coding occurred across the 4 researchers, at which point the codes and coding decisions were finalized. The final version of the codebook (Appendix B in Supplemental Materials, http://links.lww.com/HC9/A644) was then applied by one researcher per transcript to the remaining transcripts. Once the coded transcripts were reconciled, code reports were generated for each code, and narrative summaries were written, which included a narrative description of the themes and sub-themes that emerged related to each code with illustrative patient quotes highlighting each theme. Lastly, posttreatment symptoms were graded by qualitative researchers using the following severity scale: Severe: bed-ridden for days/behavior completely altered and fully prohibitive from normal routine; Moderate: had to lie down or alter behavior from the norm for a (<7 d) recovery length, challenging to daily functions but not fully prohibitive; Normal: could continue daily routine with symptom(s). We adhered to the Standards for Reporting Qualitative Research.^24^ A number of steps were taken to ensure the credibility of our research findings: use of experienced interviewers; investigator triangulation (more than 1 researcher coded the data); use of an approved interview guide; audio recording and transcribing interviews; use of qualitative software to manage the coding of transcripts; use of a common, piloted codebook; use of multiple coders; use of summary writing by multiple analysts; use of more than 1 source of data (extraction of clinical data); and persistent observation (ie, process of developing and refining the codes).^25^

## RESULTS

### Participant characteristics

Baseline characteristics of study participants (n = 26) can be found in Table 1. All patients had cirrhosis, nearly all were well-compensated (84.7% Child-Pugh A class), and half of the participants had very early (Barcelona Clinic Liver Cancer [BCLC]-0) or early stage (BCLC-A) HCC (n = 13, 50%). Eight participants (30.8%) had advanced-stage (BCLC-C) HCC due to an Eastern Cooperative Oncology Group performance status of 1 or 2. Participants received the following LRTs: TACE (n = 10, 38%), TARE (n = 6, 24%), SBRT (n = 6, 24%), and thermal ablation (n = 4, 15%) with 2 percutaneous ablations and 2 laparoscopic ablations. Interviews were performed a median of 26 days (IQR 21–33) after treatment. Supplemental Table S1, http://links.lww.com/HC9/A644 includes information on liver disease complications and other relevant diagnoses to provide context on the potential impact of liver disease severity and other comorbidities on pretreatment and posttreatment symptoms.

**TABLE 1:** Baseline characteristics of participants

Characteristic	N (%)/Median (IQR)
Age, y (median, IQR)	66.5 (61–71)
Male sex (%)	20 (76.9)
Race (%)	
Asian	1 (3.9)
Black	4 (15.4)
White	20 (76.9)
Other	1 (3.9)
Ethnicity (%)	
Hispanic	1 (3.9)
Non-Hispanic	25 (96.1)
Treatment type	
TACE	10 (38.5)
TARE	6 (23.1)
SBRT	6 (23.1)
Thermal ablation	4 (15.4)
Liver disease etiology (%)	
ALD	6 (23.1)
ALD/HCV	6 (23.1)
HBV	1 (3.9)
HCV	8 (30.8)
MASLD	5 (19.2)
Child-Pugh class (%)	
5A	14 (53.9)
6A	8 (30.8)
7B	2 (7.7)
8B	1 (3.9)
C	1 (3.9)
HE (%)	
None	21 (80.8)
Controlled	4 (15.4)
Uncontrolled	1 (3.9)
Ascites (%)	
None	20 (76.9)
Controlled	6 (23.1)
Uncontrolled	0 (0)
ECOG (%)	
0	17 (65.4)
1	5 (19.2)
2	4 (15.4)
BCLC stage (%)	
0	2 (7.7)
A	11 (42.3)
B	4 (15.4)
C	8 (30.8)
D	1 (3.9)

Abbreviations: ALD, alcohol-associated liver disease; BCLC, Barcelona Clinic Liver Cancer; ECOG, Eastern Cooperative Oncology Group; MASLD, metabolic dysfunction-associated steatotic liver disease; SBRT, stereotactic body radiation therapy; TACE, transarterial chemoembolization; TARE, transarterial radioembolization.

### Patient-reported pretreatment symptom burden

Participants were asked to describe symptoms leading up to their HCC diagnosis and the degree to which symptoms interfered with daily activities (frequency of symptoms and representative quotations in Table 2). Over half (n = 16, 62%) of patients reported no symptoms before learning about their HCC diagnosis.

**TABLE 2:** Representative quotations from patients with HCC regarding pretreatment symptoms among all participants

Symptom (frequency, %)^a^	Illustrative quotes
No symptoms experienced (n = 16, 62%)	*I didn’t feel nothing, you know? Felt all right… it was accidental basically* (male, BCLC-B)
	*Actually, I didn’t really know I was sick because I didn’t feel no different really.* (male, BCLC-C)
	*Then you know you hang this diagnosis over top of me and it’s like, “Wait, I don’t understand because I don’t feel bad.” I don’t feel sick. It was just confusing.* (female, BCLC-B)
Pain in abdomen/lower back (n = 9, 34%)	*I think possibly a slight bit more you know kind of a dull ache or kind of a very shallow sort of level of pain but just kind of a mental acknowledgment that there was something there.* (male, BCLC-A)
	*Well, the location on the liver where it was, was right under my diaphragm. It was a really difficult spot…and I guess that’s why it was so painful.* (male, BCLC-C)
	*My side was starting to hurt again as I did activities. It was hard for me to walk for any length or period.* (male, BCLC-C)
Fatigue (n = 6, 23%)	*I started noticing that I was very, very fatigued… I had to pretty much lay down and go to sleep. And I was a very active person, did not take naps in the daytime.* (female, BCLC-C)
	*Some days I would wake up in the morning and I was really, really tired and I really didn’t feel like doing anything so I’d just lay down… And I knew something was different then.* (female, BCLC-C)
Nausea (n = 3, 12%)	*I had been feeling kind of bad for like two days but I just felt like, sort of like nauseated but I guess not really nauseated.* (male, BCLC-A)
Loss of appetite (n = 2, 8%)	*And felt real full. My appetite had dropped a lot before [presenting in clinic] and it’s still down. I don’t eat hardly half of what I used to eat at a regular meal.* (male, BCLC-D)
Difficulty breathing (n = 2, 8%)	*Well, I’ve been having a lot of trouble breathing… I had run for a couple of years and wasn’t a bother but then all the sudden, I started having trouble breathing.* (male, BCLC-D)

a One patient made mention of blood in stool, confusion/memory loss, diarrhea, bloating, headaches, and discoloration of skin.

Abbreviation: BCLC, Barcelona Clinic Liver Cancer.

Among those who did report symptoms (n = 10) prior to HCC diagnosis, the most common symptoms were abdominal pain (n = 9, 35%), fatigue (n = 6, 23%), and nausea (n = 3, 12%) (Table 2). Some participants noted that the workup of a symptom (eg, abdominal pain or gas) led to their diagnosis of HCC, some were certain that symptoms were caused by liver cancer, and others expressed uncertainty about whether symptoms were attributable to liver cancer or nonliver chronic health conditions.

### Patient-reported posttreatment physical symptoms

All participants (n = 26) reported at least 1 posttreatment physical symptom. A total of 30 different symptoms were described. Of these symptoms, the majority were new symptoms, although 8 patients reported one of the same symptoms before and after treatment. Representative quotations illustrating symptoms are provided in Tables 3–6, describing symptoms after TACE, TARE, SBRT, and thermal ablation. Tables list the frequency and severity of symptoms and the frequency of symptoms present prediagnosis.

**TABLE 3:** Frequency of pretreatment and posttreatment symptoms and severity of posttreatment symptoms after TACE (n = 10)

			Posttreatment symptom severity^a^			
Symptom^b^	Pretreatment n, (%)	Posttreatment n, (%)	Normal	Moderate	Severe	Illustrative quotes and symptom severity score
Appetite loss	1 (10)	8 (80)	2	4	2	*I lost appetite and I still ain’t got my appetite back, I’m cooking a chicken right now…but I don’t eat like I used to and the things that I used to like, I don’t care much about no more.* (Normal) (male, BCLC-C)
Nausea	2 (20)	5 (50)	2	2	1	*The only thing that I could say that’s got worse is I’ve been nauseated with dry heaves on several occasions in the last week.* (Moderate) (male, BCLC-D)
Fatigue	1 (10)	5 (50)	3	1	1	*He was so worn out and he’s so tired of people and he’s so tired of everything. (caregiver)* (Severe) (male, BCLC-C)
Weight loss	0	5 (50)	4	1	—	*And I think since I was in the hospital… I was 257 pounds and I weighed myself yesterday and I am 229 now so.* (Moderate) (male, BCLC-A)
Pain						
General pain	1 (10)	2 (20)	—	1	1	*I still have that pain. And if anything, it might be a little more severe than before, because I notice it more now* (Moderate) (male, BCLC-C)
Shoulder pain	1 (10)	2 (20)	1	1	—	*It made me have that pain, that real sore pain when I take a deep breath and the same nerve that’s there runs up through my shoulder because my shoulder was hurting also at the same time.* (Moderate) (male, BCLC-A)
Dull upper right quadrant pain	0	2 (20)	2	—	—	*Pain, especially around my liver area.* (Normal) (male, BCLC-C)
Diarrhea	1 (10)	2 (20)	1	—	1	*I was in a little bit of pain, always cramping you know, had diarrhea… I had to be close to the toilet. And that lasted about five days and I still got a little discomfort.* (Severe) (male, BCLC-A)
Dizziness	0	2 (20)	2	—	—	*I was feeling dizzy.* (Normal) (male, BCLC-B)
Loss of taste	0	2 (20)	—	2	—	*Food has no taste.* (Moderate) (male, BCLC-C)
Soreness	0	2 (20)	—	2	—	*Well, right before that, I started feeling some cramps and stuff like that and soreness.* (Moderate) (male, BCLC-D)
Abdominal pain/cramping	0	1 (10)	1	—	—	*They said I was going to be sick the first five days. I mean I took nausea medicine and the pain pills and nothing. Now, the second five days, I was in a little bit of pain, always cramping.* (Normal) (male, BCLC-D)
Urinary urgency	0	1 (10)	—	1	—	*Like before, my wife was afraid to take me to the local Walmart because I’d have accidents. And from that point, it just hit me, it hit me and that was it. It was no time to blow a whistle or anything.* (Moderate) (male, BCLC-D)
Neuropathy	0	1 (10)	—	1	—	*My feet are still hurting after that three weeks. I don’t know what*…*because they never used to hurt…it’s like numbness.* (Moderate) (male, BCLC-B)
Leg cramps	0	1 (10)	—	1	—	*I had cramps in my legs, and they are pretty much gone now. But, man, I had a bout last Friday, I could barely walk.* (Moderate) (male, BCLC-C)
Shortness of breath	1 (10%)	1 (10)	—	1	—	*I was sweating real bad and I couldn’t take deep breaths. I was short of breath. And I was just real sore under my rib area.* (Moderate) (male, BCLC-A)
Headache	0	1 (10)	1	—	—	*A little dizziness, maybe a little headache, but other than that? Pretty good.* (Normal) (male, BCLC-B)

a Side Effect Severity Grade: Normal: could continue daily routine with symptom(s); Moderate: had to lie down or alter behavior from the norm for a (< 7 d) recovery length, challenging to daily functions but not fully prohibitive; Severe: bed-ridden for days/behavior completely altered and fully prohibitive from normal routine.

b Frequency of symptoms listed in order from most to least often mentioned.

Abbreviations: BCLC, Barcelona Clinic Liver Cancer; TACE, transarterial chemoembolization.

**TABLE 4:** Frequency of pretreatment and posttreatment symptoms and severity of posttreatment symptoms after TARE (n = 6)

			Posttreatment symptom severity^a^			
Symptom^b^	Pretreatment n, (%)	Posttreatment n, (%)	Normal	Moderate	Severe	Illustrative quotes and symptom severity score
Fatigue	1 (17)	3 (50)	—	1	2	*[I] don’t feel like going out…and getting up and enjoying the day, I’m just too tired. All I want to do is just lay down.* (Severe) (female, BCLC-C)
Vomiting	0	3 (50)	1	1	1	*So, I do experience some queasiness… I thought I was going to be okay and I think I tried to eat something, but nothing stayed down that entire night. Not water, not soda, not ginger ale, nothing stayed down.* (Normal) (female, BCLC-C)
Appetite loss	1 (17)	3 (50)	—	2	1	*Okay I’ve got but at that point of not really up hungry… I don’t want to take the time to stop and eat. And then I’ll start to feel a little nauseous. And I’m like okay, you know the two, the light-headed and the nausea, I’ve got to eat something whether I want it or not because it’s like the liver is a dictator and it’s bullying me, making me eat.* (Moderate) (female, BCLC-B)
Nausea	0	3 (50)	—	2	1	*I wake up in the morning and sometimes I vomit and sometimes I just gag. But it’s a very, very, like you’re going to vomit and you gag and gag and nothing comes up. And I feel that during the daytime too. I feel like, off and on all day.* (Severe) (female, BCLC-C)
Constipated	0	2 (33)	—	1	1	*I’m still struggling with getting my bowels straightened out, back to the way they were.* (Severe) (female, BCLC-C)
Gas	0	2 (33)	1	1	—	*I can hear and feel gas rumbling around in my gut. And it just - I can’t relieve it. So that’s what the pain is about. It’s all toward my you know all up in my gut is where the pains at.* (Moderate) (male, BCLC-A)
Pain						
Dull lower right pain	1 (17)	2 (33)	1	—	1	*It’s kind of odd but it’s just like a little twinge here and there. It’s not every day. It’s every once in a while.* (Normal) (female, BCLC-B)
Groin pain	0	1 (17)	—	1	—	*I do have groin pain but the groin pain usually occurs because you know what I’ve been attributing it to is because I have such a gas buildup.* (Moderate) (male, BCLC-A)
Pain/swelling at insertion site	0	1 (17)	1	—	—	*The only thing I experienced was pain from the site where they were going in.* (Normal) (male, BCLC-A)
Abdominal pain/cramping	0	1 (17)	—	1	—	*Well, my abdominal pain…the most bothersome actually is you know I guess you know the problems with my gut whether it’s indigestion or whatever.* (Moderate) (male, BCLC-A)
Dizziness	0	1 (17)	—	1	—	*It’s like if you don’t have anything working in the liver, I’ll get dizzy, light-headed and feel like I’m going to hurl.* (Moderate) (female, BCLC-B)
Trouble urinating	0	1 (17)	—	1	—	*I can’t really urinate like I should.* (Moderate) (male, BCLC-A)
Soft stool	0	1 (17)	1	—	—	*The stool has been softer than it was before.* (Normal) (female, BCLC-B)
Skin color darkened	0	1 (17)	1	—	—	*Then I noticed my skin color, and it’s gotten darker… And I don’t even leave the house.* (Normal) (male, BCLC-A)

a Side Effect Severity Grade: Normal: could continue daily routine with symptom(s); Moderate: had to lie down or alter behavior from the norm for a (< 7 d) recovery length, challenging to daily functions but not fully prohibitive; Severe: bed-ridden for days/behavior completely altered and fully prohibitive from normal routine.

b Frequency of symptoms listed in order from most to least often mentioned.

Abbreviations: BCLC, Barcelona Clinic Liver Cancer; TARE, transarterial radioembolization.

**TABLE 5:** Frequency of pretreatment and posttreatment symptoms and severity of posttreatment symptoms after SBRT (n = 6)

			Posttreatment symptom severity^a^			
Symptom^b^	Pretreatment n, (%)	Posttreatment n, (%)	Normal	Moderate	Severe	Illustrative quotes and symptom severity score
Fatigue	2 (33)	5 (83)	—	4	1	*Well, I just felt raw inside you might say and tired. Just now, I’m getting a little better on the tiredness but they said after two weeks it would start getting better you know. But as long as I’m moving and everything, I’ll be all right but if I sit down just for a minute, I drifted off like you wouldn’t believe.* (Moderate) (male, BCLC-0)
Appetite loss	0	4 (67)	3	1	—	*Yes, I feel very full when I eat and sometimes I don’t feel like eating.* (Moderate) (male, BCLC-A)
Constipated	0	2 (33)	—	2	—	*I remember not being able to pass a movement for…a few days. I mean, it was really kind of impacted.* (Moderate) (male, BCLC-B)
Nausea	0	2 (33)	1	1	—	*It was like a wave of nausea. I remember it was in the afternoon, I didn’t feel like eating that night and then I was okay the next day and then the day after that I got a wave of nausea and I didn’t take anything for it just kind of unsettled and you know I thought it might be related to the treatment.* (Moderate) (male, BCLC-A)
Pain						
Dull lower right pain	1 (17)	2 (33)	1	1	—	*Kind of a dull ache or… a very shallow sort of level of pain but just kind of a mental acknowledgment that there was something there.* (Normal) (male, BCLC-A)
General pain	0	1 (17)	—	—	1	*Initially it was the inability to move. I mean I was really pretty sore and I needed help getting up and pretty loud like, “Ahhh” out of the bed or even lying down.* (Severe) (male, BCLC-B)
Soreness	0	1 (17)	1	—	—	*No, it’s just the…the soreness you might say…you kind of feel sore, you kind of feel like you might be trying to come down with a cold, flu-like stuff, or something like that even. You know I mean your body just isn’t right.* (Normal) (male, BCLC-0)
Diarrhea	0	1 (17)	—	1	—	*Well… diarrhea was the worst thing*. (Moderate) (male, BCLC-A)
Vomiting	0	1 (17)	—	—	1	*You know it kind of exasperated the throwing up process. And that’s why I was a little bit hesitant to say you know that it was the radiation that caused it. But it kind of enhanced the throwing up. I couldn’t even get to the hospital that day I was throwing up so hard.* (Severe) (male, BCLC-A)
Bloody stool	0	1 (17)	—	1	—	*Then I start bleeding [in the stool]. One moment I start bleeding…after three, four days you know. It continued for four days.* (Moderate) (male, BCLC-A)
Swelling	0	1 (17)	1	—	—	*But other than that, I had a little bit of it felt like kind of my rib cage, like it was swollen you know. Nothing real bad, something you could live with but I felt it, felt swollen a little bit.* (Normal) (male, BCLC-A)
Fever	0	1 (17)	1	—	—	*And then I had a little bit of a fever but it was not above like 100 so I did not call the doctor because the next day I was all right, yes.* (Normal) (male, BCLC-A)
Night sweats	0	1 (17)	1	—	—	*Yeah so the first treatment, I had a little bit you know problem, sweating and that weakness.* (Normal) (male, BCLC-A)

a Side Effect Severity Grade: Normal: could continue daily routine with symptom(s); Moderate: had to lie down or alter behavior from the norm for a (<7 d) recovery length, challenging to daily functions but not fully prohibitive; Severe: bed-ridden for days/behavior completely altered and fully prohibitive from normal routine.

b Frequency of symptoms listed in order from most to least often mentioned.

Abbreviations: BCLC, Barcelona Clinic Liver Cancer; SBRT, stereotactic body radiation therapy.

**TABLE 6:** Frequency of pretreatment and posttreatment symptoms and severity of posttreatment symptoms after thermal ablation (n = 4)

			Posttreatment symptom severity^a^			
Symptom^b^	Pretreatment n, (%)	Posttreatment n, (%)	Normal	Moderate	Severe	Illustrative quotes and symptom severity score
Pain/swelling at insertion site (n = 3, 75%)	0	3 (75)	—	3	—	*And the worst pain of all of them was in my belly button where it went through. It was just irritating. And but I was in a little bit of pain for about three days and starting about the fourth day I started feeling a lot better. I didn’t have to take any more of the pain medication. (*Moderate) (male, BCLC-C)
Fatigue (n = 2, 50%)	1 (25)	2 (50)	1	—	1	*And the tiredness, I still get tired. I don’t have the energy that I should have, or someone that doesn’t have the health problems would have that I got, you know. And my motivation is not so great. Yesterday was my birthday. And I didn’t do a whole lot. I went shopping for a little while, maybe an hour, an hour and a half, and I came home, and I had a few family members to come over. And I couldn’t wait for them to leave, to be honest with you. I mean, I don’t mean that in a bad way. But I was tired, they stayed a few hours, and I got really tired.* (Severe) (female, BCLC-C)
Nausea (n = 2, 50%)	1 (25)	2 (50)	—	1	1	*Now, I did have nausea for about a week, nauseous, sick to my stomach, which they knew that and they sent me home with these pills that you put on your tongue and that calms your stomach down*. (Moderate) (male, BCLC-C)
Abdominal pain/cramping (n = 2, 50%)	1 (25)	2 (50)	—	2	—	*I was taking it easy but I still was feeling the pain like pulling pressure or whatever and I still feel some of that now.* (Moderate) (female, BCLC-A)
Pain (around liver, shoulder) (n = 1, 25%)	0	1 (25)	—	—	1	*When I was having that pain that felt like I was having my liver shredded to pieces, there was also another pain in my lower right side. And that was totally different pain. That felt like someone was taking their finger and poking me in my side really, really hard, and it was causing a sharp pain…and it was down below where my liver is, completely on the right side though, on your side not below my liver but more to the right side of my liver, but down…like towards my hip bone.* (Severe) (female, BCLC-C)
Soreness (n = 1, 25%)	0	1 (25)	1	—	—	*And I still have some soreness but when we talked the other day, he told me it’d probably be like - At that time, it was two weeks after the treatment before it’d really start kicking in.* (Normal) (male, BCLC-C)
Appetite loss (n = 1, 25%)	0	1 (25)	—	1	—	*For a while there, food, just nothing sounded good. I was eating like a bird although I don’t know why I didn’t lose weight because I’m still pretty hefty.* (Moderate) (male, BCLC-A)
Shortness of breath (n = 1, 25%)	0	1 (25)	—	—	1	*But when the shortness of the breath, it was like that lasted for about two weeks. And so I wasn’t kind of expecting that like but she did tell me that it could be that because when they blow you up you know, you’re going to feel that way.* (Severe) (female, BCLC-A)
Bleeding from wound (n = 1, 25%)	0	1 (25)	—	1	—	*Bled like hell. Saturated the gauze, the first one, changed it, saturated the second one and changed it, and then a few days it just stopped bleeding altogether. So I don’t know whether that was normal or not.* (Moderate) (male, BCLC-A)

a Side Effect Severity Grade: Normal: could continue daily routine with symptom(s); Moderate: had to lie down or alter behavior from the norm for a (< 7 d) recovery length, challenging to daily functions but not fully prohibitive; Severe: bed-ridden for days/behavior completely altered and fully prohibitive from normal routine.

b Frequency of symptoms listed in order from most to least often mentioned.

Abbreviation: BCLC, Barcelona Clinic Liver Cancer.

Nearly three-quarters of patients reported appetite loss (n = 19, 73%). This was reported after all LRTs but was particularly common and severe after TACE. Over half of participants reported posttreatment fatigue (n = 15, 58%), although this was more common and severe among participants treated with radiation-based therapies (TARE and SBRT). Pain, most often in the abdomen, was commonly reported (n = 12, 46%), although it was generally mild to moderate in severity. Participants who received SBRT experienced discomfort related to fiducial marker placement, including one who said, “You know they did describe saying you know, ‘we’re going to put these markers in,’ and I just didn’t understand they were going to be actually you know penetrating my skin and setting these markers down” (male, BCLC-A). Participants who received laparoscopic ablation reported complications from the placement of the ablation probe, including pain and bleeding. Other common symptoms after LRTs included nausea (n = 9, 35%), weight loss (n = 6, 23%), constipation (n = 4, 15%), and vomiting (n = 4, 15%).

Participants reported that the symptoms that most impacted their daily activities or routines were diarrhea, pain, and nausea. Changes in daily activities included decreased work productivity, inability to do daily exercise routines, inability to walk or take in normal activities, and ED visits for unexpected symptoms. Changes in work routine primarily came from lifting restrictions. Participants also reported their walking was limited by physical symptoms such as nausea, fatigue, and dizziness.

Eight participants (31%) had an ED visit within 3 months of treatment, and 1 patient had 2 ED visits. Five participants (19%) had inpatient hospitalizations within 3 months of LRT for gastrointestinal bleeding (n = 2), HE (n = 1), PVT (n = 1), and cerebrovascular accident (n = 1).

### Anticipation and management of posttreatment symptoms

Many participants emphasized a need for “balance” between getting too much information about a procedure, which may incite anxiety or fear, and having enough information to make an educated and informed treatment decision. Most participants felt like their providers did a good job with this balance, as indicated by the following statement, “I think I could have known too much, and that only gives you food to worry about. From my perspective, I think I knew just enough” (male, BCLC-C).

However, when asked about aspects of treatment they would improve, several participants expressed a desire to review potential side effects in-person with a member of the oncology team rather than reviewing this information in printed patient instructions.

Participants also desired more information about how to handle symptoms in a timely manner as they were encountered. Several participants indicated that no members of the care team explained what to expect after treatment, nor did they hear from anyone to check in on them after treatment. For instance, one participant shared, “They sent me home with paperwork. He said that, “You shouldn’t have much pain with this. You can take Tylenol for what you do have.” And it was, “Don’t be calling up here asking me for pain medicine,” is the way I took it. Which I wouldn’t anyway because I don’t want to take pain medication. But as far as not having an appetite and vomiting, there was no instruction on that” (female, BCLC-C).

Some participants contacted care team members through electronic medical record messaging or telephone calls due to new or worsening symptoms. This contact was often prompted by a lack of understanding about whether the care team would contact the patient directly. For instance, one participant stated, “Well, I’ve read through some of the stuff, and I was under the impression that someone from up there would contact me like a couple weeks after the procedure” (male, BCLC-A).

Some participants mentioned seeking information on the internet about potential side effects and general information about liver cancer, which often increased distress. For instance, “I want to say it was that morning, that Sunday morning I Googled that. That Monday I called and I wanted to talk to [provider] and then I spoke to the nurse and conveyed my concerns about what I had read when I Googled it because I was pretty upset from what I read. I mean to where I cried and everything and I just had to close it down and come out of there” (female, BCLC-B).

Patients described several ways in which they attempted to remedy their symptoms, such as taking medications including lactulose and rifaximin for fatigue, antiemetics for nausea, and analgesics (acetaminophen and oxycodone) for pain. Oral cannabidiol and smoking marijuana were used by 2 participants to address nausea, pain, and indigestion.

### Interest in electronic symptom monitoring using PRO instruments

Based on a description provided by the interviewers, participants were asked about their initial impressions of electronic symptom monitoring. Representative quotes are depicted in Table 7. Most participants (20/26, 77%) stated that there could be potential benefits in an electronic symptom monitoring system. Participants discussed several potential benefits of soliciting posttreatment symptoms through electronic surveys, including the potential to heighten their team’s awareness of new or worsening symptoms, improved patient-provider communication, and the ability to track symptoms over time.

**TABLE 7:** Potential benefits and challenges in electronic symptom monitoring using PRO instruments

Topic	Theme	Illustrative quotes
Potential benefits of electronic PRO monitoring	Sounds easy and straightforward	*I think that would be easy…I don’t think that would be any problem you know. I think that would be very helpful for a lot of people…I’d fill out the questionnaire but then if I was worried about something, I’d call.* (female, BCLC-0)
	May serve to heighten care team’s awareness of patient’s condition	*I think that the electronic questionnaire would be a great thing because it can get the answers back to the proper channels a lot faster. But also I think that it should be continued that someone reach out to the patient because you can get more of an understanding if you can talk to them because if you just got a questionnaire and it’s a yes or no…If you have them on the phone, you can be like, “Okay, are you experiencing this?” “No, I’m not but I did have a little of this, little of that.” You know, I think it would still be a good thing to do to talk to them personally. But as far as a quick information back to the doctor, I think the electronic would definitely work.* (male, BCLC-A)
	—	*I think it would be real helpful. I had an uncle that passed away a couple years ago. He died of liver cancer…And I can remember him telling me when I talked to him, he would tell me this was when he was going through his treatment. He was constantly telling me that his stomach was killing him. He said, “I hurt all the time”* (male, BCLC-A)
	May provide opportunity to track symptoms over time	*It sounds like a, you know, good idea. Like I said, occasionally I still have some side effects from that. So it would be good to keep it, you know, recorded, wrote down, you know, keep track of them. That could be helpful.* (female, BCLC-C)
	May help to improve patient-provider communication in general and specifically during a clinic visit	*Oh, it would. I mean, it would give the doctor some background when you get there because sometimes when you get there you don’t remember, you always forget to say something.* (male, BCLC-0)
	May increase the feeling among patients that someone is listening to them and they feel more connected to their care team	*For me to know that someone out there is listening, you know, that they’re seeing and they’re taking under consideration what I’m going through, you know, what happened with me and what’s still happening with me. That would give me peace of mind, you know. Yeah, I would be happy to know that someone cares. And if by doing that, you know, I’m like, well I’m not doing this for nothing.* (female, BCLC-C)
	May provide patients with a sense that they are not alone in their recovery	*This would be actually very good. If it helps someone, you know they can find out this is like if you have ten or twenty people that have the same problems so.* (male, BCLC-A)
	May provide patients with a chance to help other patients	*They’re definitely going to see it, they’re definitely going to read it. And they’re definitely going to record it, so it may help somebody else down the line, you know so they could maybe do something different or maybe find a way to tell who’s, you know, who’s going to be able to do this procedure without this pain that I went through, you know, and who may not because right now, they don’t have any way of knowing that.* (female, BCLC-C)
Potential limitations or downside of electronic PRO monitoring	Self-described “non questionnaire” kind of patient	*I’ve never had any problem calling my doctor and telling him I had these side effects. And so I mean it’s basically the same principle I think.* (male, BCLC-A)
	Concern the ePRO responses will be redundant and not reveal anything of value	*Yeah I mean because you could just set aside one day a week to do it I mean but unless people are experiencing more side effects than I am, I don’t know like mine would be redundant. It would be the same thing all the time…I mean if you don’t have anything to input, why would you answer the questionnaire? Wouldn’t it be redundant? Wouldn’t it be the same thing every week?* (female, BCLC-B)
	Concern ePROs will be too general and not be applicable to the individual patient	*So, it’s all how you feel…and yet your feelings might not have really nothing to do with what I just went through but because it’s got to be so general, that’s what I don’t like about surveys.* (male, BCLC-D)
	Why complete the ePRO when I can call my doctor?	*But, I mean, the answers are going to be the same. I think that the doctor was very informative, told me how I was going to feel, asked me if I had any questions…I think that what he said, and he covered every angle of it while I was in there, there was nothing that I felt like that he would not do if I called him and asked him. Even the doctor that was with him came in at the end and said, “If you have any problems at all, I want you to call me.”* (female, BCLC-C)
	Concern over the patient mental burden from receiving a recurring ePRO	*Right now, I’m neutral with not having seen it. If it’s a burden on someone who’s depressed, it’s not helpful. If he’s healthy in mind and in body, especially in mind, it may be a good tool. The thing is not to rule out anything that you’re trying to encourage the patient to get well.* (male, BCLC-C)
	Concern no one will see or care about the questionnaire responses	*When you get to talking to somebody that really … you hear a voice on the end of the phone, and it makes you feel better being able to talk about it, gives you some relief. Knowing that somebody out there is concerned enough to know what’s going on with you. Yes, so it means a lot, but to sit down and just fill out a questionnaire. What do you think it, you know? Who’s going to really see this? It could be somebody out there that’s really going to see this. But while you’re doing it, you don’t feel like it’s going to have that much of an impact, or you don’t feel like it’s going to help anybody else if it’s not you.* (male, BCLC-A)

Abbreviations: BCLC, Barcelona Clinic Liver Cancer; PRO, patient-reported outcomes.

Potential limitations or downsides included concern that the electronic surveys would be unnecessary if treatment was uncomplicated, that surveys would be too general and not specific enough for individual patients, and concern about the mental burden placed on patients required to complete the surveys. Furthermore, some patients stated that surveys may be unnecessary since they had no problems calling their care team to report symptoms.

Most (n = 18, 69%) participants were in favor of an online tool that would allow them to fill out electronic PRO surveys using a smartphone or electronic device (eg, laptop). Some patients (n = 6, 23%) expressed a preference for telephone-based symptom monitoring due to a lack of computer literacy or access to a smartphone or computer. Most patients (n = 21, 81%) were confident they could complete symptom questionnaires without assistance. Regarding the frequency of completing surveys, there were a range of patient responses, including willingness to submit weekly, biweekly, or monthly electronic surveys. Other participants suggested that the frequency should be flexible and depend on factors such as time to accommodate variability in posttreatment symptom timing and severity.

## DISCUSSION

Using qualitative research methods to elicit patients’ experiences in their own words, this qualitative study of patients with HCC recently receiving LRTs demonstrates that while pre-HCC diagnosis symptoms are reported by a minority of patients, posttreatment symptoms are universal and lead to a variety of impairments in daily activities and functioning. Posttreatment symptoms result in increased health care utilization, with nearly one-third of participants requiring an ED visit within 3 months of undergoing treatment. Patients described diarrhea, pain, and nausea as symptoms that interfered most with daily activities and functioning. Interference with daily activities and the frequency of posttreatment health care utilization demonstrates the impact these symptoms have on their lives and highlights opportunities to enhance patient-centered care. Participants expressed interest in electronic symptom monitoring, with the majority preferring a web-based format and completing surveys weekly or biweekly. These findings suggest that electronic symptom monitoring in patients after HCC treatment may be an acceptable means to proactively address patient-reported symptoms. A symptom monitoring system may have the capacity to improve patients’ functioning and quality of life after LRT treatment and reduce health care utilization.

Unlike prior qualitative studies in HCC, our study includes perspectives from patients who have received SBRT, PRO data collected on average 3 weeks after LRT treatment, and specifically evaluated patient perspectives on the role of electronic symptom monitoring. Our study adds several unique aspects to this literature, including elucidation of novel symptoms not highlighted in prior work (eg, nausea, constipation, loss of taste, weight loss, and pain/swelling/bleeding from wounds).

### Patient-reported symptoms to inform selection of PROs most relevant to patients’ quality of life

Our findings have the potential to improve HCC care in several ways. A better understanding of posttreatment patient-reported symptoms will ensure the selection of PRO measures that quantify symptoms, most importantly from the patient's perspective. The importance of PROs as meaningful treatment outcomes has been recognized by the US Food and Drug Administration^9^ and has led to the inclusion of PROs in recent clinical trials of treatments for advanced-stage HCC.^26–30^ Future comparative effectiveness and toxicity studies among patients receiving LRT that incorporate appropriate PROs will directly inform patient-provider communication and resulting treatment decisions. In a large survey study, most patients with HCC preferred treatments that maximized overall survival but were willing to accept tradeoffs in survival to avoid treatment-related side effects.^31^ This may be particularly relevant for noncurative LRT options, which often have similar effects on overall survival but distinct side effect profiles. Thus, real-world data incorporating relevant PROs will inform decisions for patients and clinicians.

### Need for HCC-specific PRO instruments among early/intermediate-stage patients with HCC

It is not clear which PRO instrument or combination of instruments is best equipped to monitor disease-related or treatment-related symptoms in HCC, although there is emerging guidance in cirrhosis.^32^ There are several PRO instruments that have been empirically validated that could be used to monitor HCC-related or treatment-related symptoms in this population.^12^ Generic instruments, such as the European Organization for Research and Treatment of Cancer (EORTC) quality of life questionnaire -C30,^33^ Short Form 36,^34^ or Short Form -8, have been empirically validated but currently lack content validity for patients specifically living with HCC. Disease or treatment-related symptoms may be more thoroughly assessed by disease-specific instruments, such as the EORTC quality of life questionnaire-HCC-18,^35^ Functional Assessment of Chronic Illness TherapyHepatobiliary,^36^ or the Functional Assessment of Chronic Illness Therapy Hepatobiliary Symptom Index-8.^37^ The Hepatobiliary Symptom Index-8 demonstrated good psychometric properties among patients with HCC^37^ and has established meaningful change scores^30,38^ but may not include all relevant symptoms for patients with localized HCC who have received LRTs. The Patient-Reported Outcomes Measurement Information System Profile-29 instrument has recently demonstrated validity and efficiency for assessing HRQOL in patients with chronic liver disease.^39^ Specific items from the cancer-specific Patient-Reported Outcomes version of the Common Terminology Criteria for Adverse Events may capture additional symptoms and treatment side effects, but more content and empirical validation are needed. The findings of our study support the need to develop new PROs specific to patients with HCC receiving LRTs. To maximize the clinical impact of any posttreatment PRO instrument, it will be important to balance the need to capture all symptoms meaningful to patients while avoiding excessive burden of patients and providers that would limit its uptake in real-world practice.

### Role of proactive symptom monitoring in HCC

Proactive symptom monitoring with electronic PRO surveys has the potential to facilitate the early identification of symptoms that can be addressed by clinicians. Proactive symptom monitoring with PRO surveys has been shown to improve physical function, symptom control, HRQOL, and decrease health care utilization and overall mortality.^14–16^ While these potential benefits are remarkable, it will be important to determine if these findings translate to localized HCC. Patients with localized HCC are distinct from those with metastatic cancers in several ways, including the presence of underlying cirrhosis, which contributes to symptoms, and less frequent contact with patients after LRTs compared to those on systemic therapy. Therefore, it is possible that patients with intermediate-stage HCC may experience larger benefits from electronic symptom monitoring. Findings from our study suggest that an electronic symptom monitoring tool has potential appeal for patients with HCC; however, the range of responses we elicited suggests the need for multiple modality options based on individual patient’s comfort level, access to internet-based technology, and need for assistance.

Furthermore, there is a need for customization to implement PROs into clinical practice. A recent concise review outlines several important factors in the process of PRO collection within clinical hepatology, including clinician training, monitoring systems to track PRO collection (eg, with integration in the electronic medical record ), and the importance of a physician champion.^40^ Future research is needed to assess when and how best to implement PROs in hepatology care and evaluate how they influence clinical care.

### Alternative strategies to address symptoms in HCC

Proactive symptom monitoring with electronic PRO instruments is just one of many potential strategies to address symptom burden in patients with HCC. A recent AASLD guidance provides an overview of palliative care and symptom-based management strategies for patients with decompensated cirrhosis.^41^ There are a number of studies demonstrating symptom burden and HRQOL can be improved in patients with decompensated cirrhosis^42–44^ and cancer^45–47^ through a number of palliative care interventions including nurse-led, psychologist-led, social worker-led, or physician-led symptom assessments, care coordination interventions, self-management interventions, and mindfulness-based stress reduction. Recently, a single-center pilot trial among patients with HCC demonstrated that visits with a palliative care provider resulted in improvements in HRQOL and symptoms.^48^

### Potential limitations

Our study is strengthened by a comprehensive qualitative assessment of posttreatment symptoms among patients with HCC who recently received LRT. However, it is important to consider its potential limitations. First, this is a single-center study limited to English-speakers who were mostly White and non-Hispanic; as such, our findings may not be generalizable to non-White, non–English-speaking patients. Of note, the UNC North Carolina Basnight Cancer Hospital serves all 100 counties of NC, and our cohort included broad representation across the state. Second, while it is difficult to determine if posttreatment patient-reported symptoms are attributable to cirrhosis, HCC, other comorbidities, or the treatment itself, the pretreatment symptoms serve as a reference for comparison. Thus, change in symptoms from pretreatment to posttreatment is presumably caused by treatment, as LRT was the main intervening factor; however, other causes cannot be ruled out. Many pretreatment symptoms could be attributable to ascites, HE, medications (eg, lactulose), and non–liver-related comorbidities (eg, diabetes mellitus, obstructive sleep apnea, gastroesophageal reflux disease) (Supplemental Table S1, Supplemental Digital Content 1, http://links.lww.com/HC9/A644). Most of our cohort had well-compensated cirrhosis with a low burden of ascites and HE, reducing the likelihood that high baseline symptoms were only due to cirrhosis. Third, while our sample did include a good distribution of LRT modalities, the sample size was insufficient to make comparisons of posttreatment symptoms stratified by specific modalities. Therefore, larger comparative studies of posttreatment symptoms and HRQOL are needed for patients with HCC receiving LRTs. Lastly, this qualitative study did not provide information on how best to guide symptom monitoring, such as whether it should be universally offered or based on baseline risk factors (eg, cancer stage or Child-Pugh score) and whether it should be patient-initiated or provider-initiated.

In conclusion, this qualitative study of patients with HCC who had recently received LRTs identified many different physical symptoms that affected daily activities and often led to ED visits. By further elucidating the symptom burden of patients following LRT, this study sets the stage for the selection of PROs for future patient-centered outcomes research. Patients expressed interest in an electronic PRO monitoring system that could be used to monitor and proactively address posttreatment symptoms. Given its demonstrated benefits in patients with metastatic cancers, electronic PRO monitoring should be studied in patients with HCC.

## Supplementary Material

SUPPLEMENTARY MATERIAL
