# Supplementary material for: Patient-reported symptoms and interest in symptom monitoring in HCC treated with locoregional therapies: A qualitative study
Source: Hepatol Commun. 2023 Nov 6;7(11):e0315. doi: 10.1097/HC9.0000000000000315 (PMC10629737; doi:10.1097/HC9.0000000000000315)
Supplement: SUPPLEMENTARY MATERIAL [file hc9-7-e0315-s001.docx]

**Supplemental Materials**

Supplemental Table 1. Liver disease severity and other relevant diagnoses for participants in qualitative interviews

|  | **Ascites** | **Hepatic encephalopathy** | **Other Relevant Diagnoses Pre-LRT** | **Treatment Type** | **Pre-Treatment Symptoms** | **Post-Treatment Symptoms** |
| --- | --- | --- | --- | --- | --- | --- |
| 1 | Controlled | Controlled | type 2 diabetes, worsening ascites, hepatic encephalopathy on lactulose | TACE | none | appetite loss, nausea, fatigue, abdominal pain, loss of taste |
| 2 | None | None | type 2 diabetes | Segmental TARE | none | pain/swelling at insertion site |
| 3 | None | None | prostate cancer s/p prostatectomy complicated by constipation | TACE | none | appetite loss, fatigue, leg cramps |
| 4 | None | None | type 2 diabetes (poorly controlled) | TACE | none | fatigue, dizziness, neuropathy |
| 5 | None | None | type 2 diabetes, heart failure with preserved ejection fraction | TACE | abdominal pain | nausea, weight loss, abdominal pain |
| 6 | None | None | epidural abscess treated with drainage and antibiotics | Segmental TARE | none | nausea, vomiting, gas |
| 7 | Controlled | Controlled | bipolar disorder, HE on lactulose | Segmental TARE | fatigue, appetite loss, abdominal pain, headache | fatigue, nausea, vomiting, appetite loss, constipation, abdominal pain |
| 8 | None | None | ongoing alcohol use disorder | TACE | none | appetite loss, fatigue, weight loss, dizziness, headache |
| 9 | None | None | none | Lobar TARE | none | nausea, appetite loss, abdominal pain, dizziness, soft stool |
| 10 | None | None | none | TACE | none | appetite loss, diarrhea, loss of taste, abdominal pain/cramping |
| 11 | Controlled | Uncontrolled | hepatic encephalopathy on lactulose and rifaximin, diuretic use for ascites, type 2 diabetes | TACE | fatigue, appetite loss, abdominal pain, bloating, diarrhea, shortness of breath, confusion/memory loss | fatigue, appetite loss, abdominal pain, nausea, weight loss, diarrhea, soreness, urinary urgency |
| 12 | None | None | gastroesophageal reflux disease | TACE | abdominal pain, nausea, shortness of breath | shoulder pain, nausea, appetite loss, weight loss, soreness, shortness of breath |
| 13 | None | None | none | SBRT | none | appetite loss, nausea, abdominal pain, diarrhea |
| 14 | None | None | bipolar disorder | TACE | nausea, abdominal pain | nausea, appetite loss, weight loss |
| 15 | Controlled | Controlled | hepatic encephalopathy on lactulose | Percutaneous Ablation | none | pain/swelling at insertion site, fatigue, soreness, appetite loss, bleeding from wound |
| 16 | None | None | coronary artery disease, ischemic cardiomyopathy with reduced ejection fraction | Segmental TARE | none | fatigue, appetite loss, constipation, gasgroin pain, abdominal pain/cramping, trouble urinating |
| 17 | None | None | rheumatoid arthritis | TACE | abdominal pain | abdominal pain |
| 18 | None | None | type 2 diabetes, coronary artery disease | SBRT | none | fatigue, appetite loss, constipation, bloody stool, fever, night sweats |
| 19 | None | None | none | SBRT | none | fatigue |
| 20 | None | None | type 2 diabetes, ongoing alcohol use | SBRT | fatigue, abdominal pain | fatigue, appetite loss, constipation, abdominal pain |
| 21 | Controlled | None | none | Laparoscopic Ablation | none | pain/swelling at insertion site, abdominal pain/cramping, shortness of breath |
| 22 | Controlled | None | chronic obstructive pulmonary disease, coronary artery disease, internal hemorrhoids | Percutaneous Ablation | fatigue, abdominal pain, blood in stool | fatigue, abdominal pain/cramping, shoulder pain |
| 23 | None | None | none | SBRT | abdominal pain, fatigue | abdominal pain, fatigue, appetite loss, nausea, soreness |
| 24 | None | None | gastroesophageal reflux disease, obstructive sleep apnea | Laparoscopic Ablation | abdominal pain, nausea, fatigue | pain/swelling at insertion site, nausea |
| 25 | None | None | type 2 diabetes, osteoarthritis | SBRT | none | fatigue, vomiting, swelling |
| 26 | None | Controlled | type 2 diabetes, chronic back pain | Segmental TARE | none | fatigue, vomiting, skin discoloration |

**Appendix A. Post-LRT in HCC Qualitative Study: Interview Guide**

**Greeting:** Hello, my name is _________ and I work with the University of North Carolina at Chapel Hill. I really appreciate you taking the time to participate in this interview.

**Purpose:** You are being asked to participate in this study because you have been diagnosed with liver cancer and have received a liver-directed treatment. I am contacting you because you indicated to your doctor and the study coordinator that you would like to participate. This information will help assist us in better understanding the different kind of symptoms and side effects patients can experience after liver cancer treatments.

**Confidentiality and Introduction:** As a reminder, I’d like to stress that our team will keep everything said here today confidential. Your name will not be connected to what you say or your research file. Your doctor will not be told what you said. I hope that you will feel free to speak openly.

Please know that there is no right or wrong answer to these questions. My goal as an interviewer is to have you feel comfortable sharing your experiences, impressions, and beliefs to help us learn how to improve access to quality medical care for others diagnosed with liver cancer. Our discussion today will last about 60 minutes.

Before we begin, I would like to state that the conversation is being audio-taped to help us remember what is said during this discussion. You may ask me to turn off the recorder at any time or simply say you do not want to answer a question.

Do you have any questions before we begin?

**Section I: Establishing context for discussion of liver cancer treatment (5 minutes)**

1. To start, tell me about when you were diagnosed with liver cancer.
   1. When did you and your health care team start the conversations about treatment options?
   2. What treatment options were presented?
   3. How were they presented to you?
   4. What treatment option did you choose?
   5. How did you decide on that option?

**Section II: Pre-treatment symptoms (5 minutes)**

1. **KEY QUESTION: What symptoms, if any, did you experience leading up to your liver cancer treatment?**
   1. [*For each symptom mentioned explore the feeling the symptom produced*]
   2. [*If more than one symptom mentioned*] Which symptom was the most bothersome?
   3. How did these symptoms interfere with your usual or daily activities?

**Section III: Post-treatment side effects (30 minutes) – Most important section**

1. **KEY QUESTION: What side effects, if any, did you experience after treatment? Keep in mind, this is likely something new or different that you did not experience before your treatment. [*if none, skip to question 8*]**

[*For each new side effect mentioned explore the feeling the side effect produced*]

1. When did these side effects start?
   1. How long did these side effects last?
   2. What things did you do to help make this better or help you feel better?
2. [*If more than one side effect mentioned*] Which of these side effects was most bothersome?
3. How did these side effects interfere with your usual or daily activities?

6a. If you had a symptom from the disease or side effect from the treatment that you needed assistance with, how would you contact your doctor? (i.e., via phone, MyChart, online, call, etc.)

1. **For the symptoms (of the disease) you were already experiencing before treatment (eg, feeling tired, no appetite) How, if at all, did these symptoms change after receiving treatment for your liver cancer?**
   1. [*If nothing offered, can prompt*:] Overall, did your symptoms get worse, improve or stay the same?
      1. To what degree do you think these changes were related to the treatment?
2. **What kind of emotions or feelings did you have going through this treatment?**
   1. You mentioned experiencing (list side effects) during treatment. How did these feelings impact those side effects?
3. **KEY QUESTION: How did you address symptoms of the disease or side effects from the treatment that were present after your liver cancer treatment?**
   1. Which symptom(s)/side effect(s) prompted you to reach out for assistance?
   2. Who did you reach out to?
   3. What advice were your given to address your symptoms?
   4. What did you think about the advice given to you?
   5. Did you follow this advice?
   6. How did your symptoms/side effects change, if at all, after this advice?
   7. What type or level of symptoms from the disease or side effects of the treatment you would need to experience before you felt the need to discuss them with your doctor?

*If patient did not reach out for help:*

1. Tell me more about why you did not seek help for your symptoms and/or side effects.
2. What steps could your medical team have taken to help address your symptoms and/or side effects?

**Section IV: Attitudes towards electronic patient reported outcomes (7 minutes)**

We are interested in your opinion on patient-reported questionnaires about symptoms/side effects. Electronic patient reported outcomes, or ePROs, allow patients to directly report their experience with symptoms/side effects through the telephone, online or smartphone apps. ePROs might allow members of the medical team to more quickly and effectively address new or worsening symptoms/side effects that arise from liver disease, liver cancer or cancer treatments.

**14). How helpful would it be if you were able to use your phone, a form on a website or an app to fill out symptom questionnaires after your treatment for your doctor to review?**

a. What would you benefit from filling out a form about your symptoms?

1. What assistance, if any, would you need in filling out symptom forms?

[If yes] What kind of help would you need?

[If yes] Who would you ask to help you?

1. How often would you be willing to fill out symptom forms: [once a week, monthly, or when at clinic]
2. What would be the best way to fill out these symptom forms: on the internet, on the phone or one paper.

**Section V: Attitudes Towards Treatment (8 minutes)**

**15) How did your post-treatment symptoms change your thoughts about going through the treatment? [*skip if no post-treatment symptoms*]**

1. How has your experience with the treatment impacted your thoughts about pursuing treatment in the future?
2. In hindsight, what things do you wish you would have known before the treatment to better address your symptoms?
3. Would you do this treatment again?
4. If you had a friend or love one who was given the same treatment as an option, what advice would you give them?

**Section VI: Closing (2 min)**

16) Is there anything else that we did not cover that you would like to share with us?

Thank you for your time today and for your willingness to participate in this interview. You will be receiving a $50 gift card in the mail for your participation.

**Appendix B. Post-LRT in HCC Qualitative Study: Codebook**

| **Code Concept** | **Code Label** | **Code Definition** |
| --- | --- | --- |
| **Section 1. Context and Background** | | |
| Context for when and how patients were diagnosed with liver cancer | **Background Diagnosis** | Comments from participants about when they were diagnosed with liver cancer and how they were diagnosed. |
| Context about treatment option conversations with provider and how they were presented | **Treatment Presentation** | Comments from participants about the kind of conversations they remember having with their provider about treatment options and the kind of information presented to them about treatment options. Use the code any time a participant recalls being presented treatment options from their provider/care team, in the past or more recently. |
| What option they chose and how they decided | **Treatment Decision** | Comments from participants on the treatment decision they chose from their option, how they decided on their treatment option, and what they prioritized in making the decision. |
| **Section 2. Pre-Treatment Symptoms** | | |
| What symptoms patients experienced pre-diagnosis and how it impacted daily life. | **Pre-diagnosis Symptoms** | Use this code when participants describe the symptoms they experienced leading up to their liver cancer diagnosis and treatment, which symptom stood out as the most bothersome and how any symptoms they experienced interfered with their daily activities. This will include any discussion about the mental or emotional state they were in prior to being diagnosed with liver cancer. |
| **Section 3. Post-Treatment Side Effects** | | |
| Any side effects from the liver cancer treatment. | **Post-Tx Side Effects** | Use this code when participants describe the side effects they experienced due to their liver cancer treatment, when the side effects started, how long they lasted, and which side effects they deemed most bothersome. |
| Degree side effects impacted daily function and whether steps were taken to alleviate the SE/get more information from health providers | **Post-Tx Side Effects Alleviate** | Use this code when participants discuss what, if anything, they did to alleviate side effects and how the side effects interfered with their daily activities.  Also use when they discuss which side effects they sought help with, who they reached out to, what advice was given, to what degree they followed the advice, and whether the advice worked to ease/alleviate side effects. |
| Emotional aspect of going though treatment | **Post-Tx Emotions** | Use this code when participants discuss the kind of emotional or psychological aspects they felt going through liver cancer treatment (i.e., before, during or after treatment). This can include how, if at all, these feelings impacted their side effects or their daily life. |
| Severity of side effect that would deem it necessary to call or discuss with their provider | **Care Team Communication** | Use this code when participants discuss any suggests or advice they received from their cancer care team regarding feedback on what to expect during and after the treatment procedure.  Also use if they responded to the hypothetical situation of what level or type of side effects from the treatment they would need to experience before they felt the need to reach out and/or discuss it with their provider.  Also use this code if participants discussed why they did not seek help from their provider/care team for their side effects.  NOTE: This code may be double-coded using the Post-Tx Side Effects Alleviate if they discuss what recommendations their care team had to directly alleviate post-tx side effects. |
| **Section 4. Attitudes about ePROs** | | |
| Any value or benefit to ePROs | **Impression of ePROs** | Use this code when participants discuss their level of experience, if any, using ePROs.  Also use this code when they discuss how helpful/unhelpful they imagined it would be to complete an ePRO questionnaire re: symptom or side effect management or the value or benefit they see in completing a questionnaire. |
| How to receive and complete ePRO | **Delivery of ePROs** | Use this code when participants discuss what would be the best way for them to complete an ePRO and if they would need help completing the ePRO, what kind of help would they need and who would they likely ask for help. |
| How often to complete ePRO | **Frequency of ePROs** | Use this code when participants discuss how often they would be willing to complete a ePRO questionnaire. |
| **Section 5. Attitudes about Treatment** | | |
| Overall treatment impressions and whether they would do it again. | **Treatment Impressions** | Use this code when participants discuss how their post-treatment side effects changed their thoughts about going through treatment, and to what degree it impacted their thoughts about pursuing treatment in the future.  Also use this code when they discuss whether they would go through the liver cancer treatment again and if they had a friend or loved one who was given the same treatment as an option, what kind of advice would they give them. |
| Changes or things they wished they had known beforehand | **Treatment Changes** | Use this code when participants discuss any things they wished had been different about their treatment experience or things they wished they had known before the treatment to better address side effects. |
